# High-speed X-ray imaging of the Leidenfrost collapse

**DOI:** 10.1038/s41598-018-36603-w

**Published:** 2019-02-07

**Authors:** Paul R. Jones, Chihpin (Andrew) Chuang, Tao Sun, Tom Y. Zhao, Kamel Fezzaa, Juan C. Takase, Dileep Singh, Neelesh A. Patankar

**Affiliations:** 1 Department of Mechanical Engineering, Northwestern University, 2145 Sheridan Road, Evanston, IL 60208 United States; 2 Argonne National Laboratory, Lemont, IL 60439 United States; 3 Advanced Photon Source, Argonne National Laboratory, Lemont, IL 60439 United States

## Abstract

The Leidenfrost layer is characterized by an insulating vapor film between a heated surface and an ambient liquid. The collapse of this film has been canonically theorized to occur from an interfacial instability between the liquid and vapor phases. The interfacial instability alone, however, is insufficient to explain the known influence of the surface on the film collapse process. In this work, we provide visual evidence for two key mechanisms governing the film collapse: the interfacial instability, and the nucleation of vapor upon multiple non-terminal liquid-solid contacts. These results were obtained by implementing high-speed X-ray imaging of the film collapse on a heated sphere submerged in liquid-water. The X-ray images were synchronized with a second high-speed visible light camera and two thermocouples to provide insight into the film formation and film collapse processes. Lastly, the dynamic film thickness was quantified by analysis of the X-ray images. This helped assess the influence of surface roughness on the disruption of the film. The results of this work encourage further investigation into non-linear stability theory to consolidate the role of the surface on the liquid-vapor interface during the film collapse process.

## Introduction

Nucleate boiling is an efficient mode of heat transfer from immersed surfaces^1–5^. At temperatures elevated beyond the nucleate boiling regime, a vapor film known as the Leidenfrost layer will form adjacent to the surface. This film is detrimental to heat transfer because of the low thermal conductivity of the vapor^1,6–8^. To promote nucleate as opposed to film boiling, it is advantageous to design surfaces that delay the occurrence of film boiling by destabilizing the Leidenfrost layer.

A significant number of experiments have been done to explore mechanisms involved in the destabilization of the Leidenfrost layer^8–22^. These experiments are generally based on sessile or impacting water droplets above heated surfaces, where the evaporation time of the droplets is measured^8–13^. When the Leidenfrost layer is formed underneath the droplet, the droplet will levitate on a self-generated vapor film above the surface. Formation of the insulating Leidenfrost layer is identified by the maximum evaporation time of the droplet as a function of increasing superheat. These experiments are often accompanied by a high-speed camera to observe droplet dynamics^8–13^. The droplet dynamics are directly influenced by the height and velocity at which the droplet is initially released, along with the size of the droplet. These parameters are often characterized by a Weber number, which typically varies across reported experiments^8–20^.

Leidenfrost droplets have also been studied using high-speed imaging of laser interference patterns^21,22^. This method is able to quantify variations in film thickness across the entire droplet, but is not able to provide an absolute measurement of the film thickness.

A second set of experiments involve observations of heated surfaces submerged in a pooled liquid^14–20^. Pool boiling experiments are fundamentally different from the evaporating droplet experiments in that there is no longer an isolated droplet, and that the effect of air saturation within the surface texture will be significantly reduced^23^.

In each set of experiments, surface texture and wettability influenced the temperature at which the Leidenfrost film collapsed^8–14,24^, yet the primary mechanisms governing the destruction of the film are still debated^1,7^. This is because it is difficult to experimentally observe the dynamics of the film collapse process across multiple time and length scales, as well as to isolate the effects of individual mechanisms.

The Rayleigh-Taylor interfacial instability is often proposed as a primary candidate for film collapse. This mechanism, described by linear stability analyses, occurs due to the instability of an interface between two fluids of different densities^7,19,25^. Other proposed mechanisms include the vaporization of water due to heterogeneous/homogeneous nucleation^1,7,19^, vaporization due to the surface temperature exceeding the liquid spinodal limit^1,7^, or the influence of temperature-dependent wettability^1,7,26^.

Knowledge of which mechanisms dominate the film collapse process will help researchers design surfaces that increase the Leidenfrost temperature and extend the more efficient nucleate boiling regime to higher superheats. The purpose of this study was to provide direct visual data to deduce mechanisms governing the film collapse process. This was accomplished by synchronizing a high-speed X-ray camera with a high-speed visible light camera and a thermocouple reader to observe destruction of the Leidenfrost film. To eliminate the destabilizing effect of gravity present in Rayleigh-Taylor interfacial instabilities, we imaged the bottom surface of a heated sphere. In this configuration, the direction of the gravitational force stabilizes the vapor film, allowing us to obtain evidence of non-gravity based interfacial instabilities, along with vapor nucleation due to liquid-solid contact. This experiment captured the entire film formation and collapse process, observed the micron-scale film collapse – connecting this information with the macroscopic behavior observed with the naked eye, and quantified the film thickness as a function of time. The following sections provide details of the experimental setup, along with an analysis of the film thickness to yield insight into the competing mechanisms responsible for the vapor film collapse.

## Experimental Methods

Two stainless steel spheres (grade 440C) and the bottom surface of one K-type thermoprobe were used as samples across multiple trials. One sphere (mass 1.03 g, 6.34 mm diameter) possessed a polished finish, while the second sphere (mass 1.02 g, 6.32 mm diameter) was coarsely roughened using sandpaper. The thermoprobe (3.1 mm probe width, 5.6 mm diameter of probe tip) possessed a smooth finish. See Table 1 and Figs S1–S3 for profilometer surface roughness measurements of the spheres. The spheres were initially cleaned using acetone before  a K-type thermocouple was spot-welded to the bottom surface of the spheres. The samples were immersed in water contained within a borosilicate test tube open to the atmosphere. The test tube was placed within helical heating coils to elevate the temperature of the samples. An Ambrell EasyHeat 2.4 kW power supply heated the samples (electrically conductive objects) using electromagnetic induction. Samples were heated until a stable film of vapor encompassed the entire surface. After this occurred, the power supplied to the samples was terminated and the system cooled naturally.

**Table 1:** Profilometer characterization of spheres.

Surface	Camera lens	Height of peaks (Ra) (*nm*)	Spacing between peaks (Rsm) (*μm*)
Smooth	10x	33.8 ± 16.6	2.1 ± 1.0
	50x	14.5 ± 8.5	1.6 ± 1.0
Rough	10x	138.5 ± 128.2	3.4 ± 3.3
	50x	42.2 ± 52.5	0.4 ± 0.5

The entire heating and cooling process was captured by a high-speed phase-contrast X-ray imaging system at the 32-ID-B beamline at the Advanced Photon Source facility at Argonne National Laboratory. In this experiment, X-ray imaging was well-suited for highlighting differences in transmitted light, which corresponded to fluid-fluid and solid-fluid interfaces. To achieve this, a monochromatic X-ray was guided using an undulator with a period of 1.8 cm, and 24 mm gap to generate X-ray photons with first harmonic energy of 25.7 keV. The transmitted photons were then projected onto a 100 *μm* LuAG:Ce (Ce-doped Lu3Al5O12 single crystal) scintillator. The visible light generated by the scintillator was reflected by a mirror and recorded by an X-ray image detector (FASTCAM SA-Z type 2100K-M-32GB). A more detailed description of the system can be found in ref.^27^.

**Figure 1:**
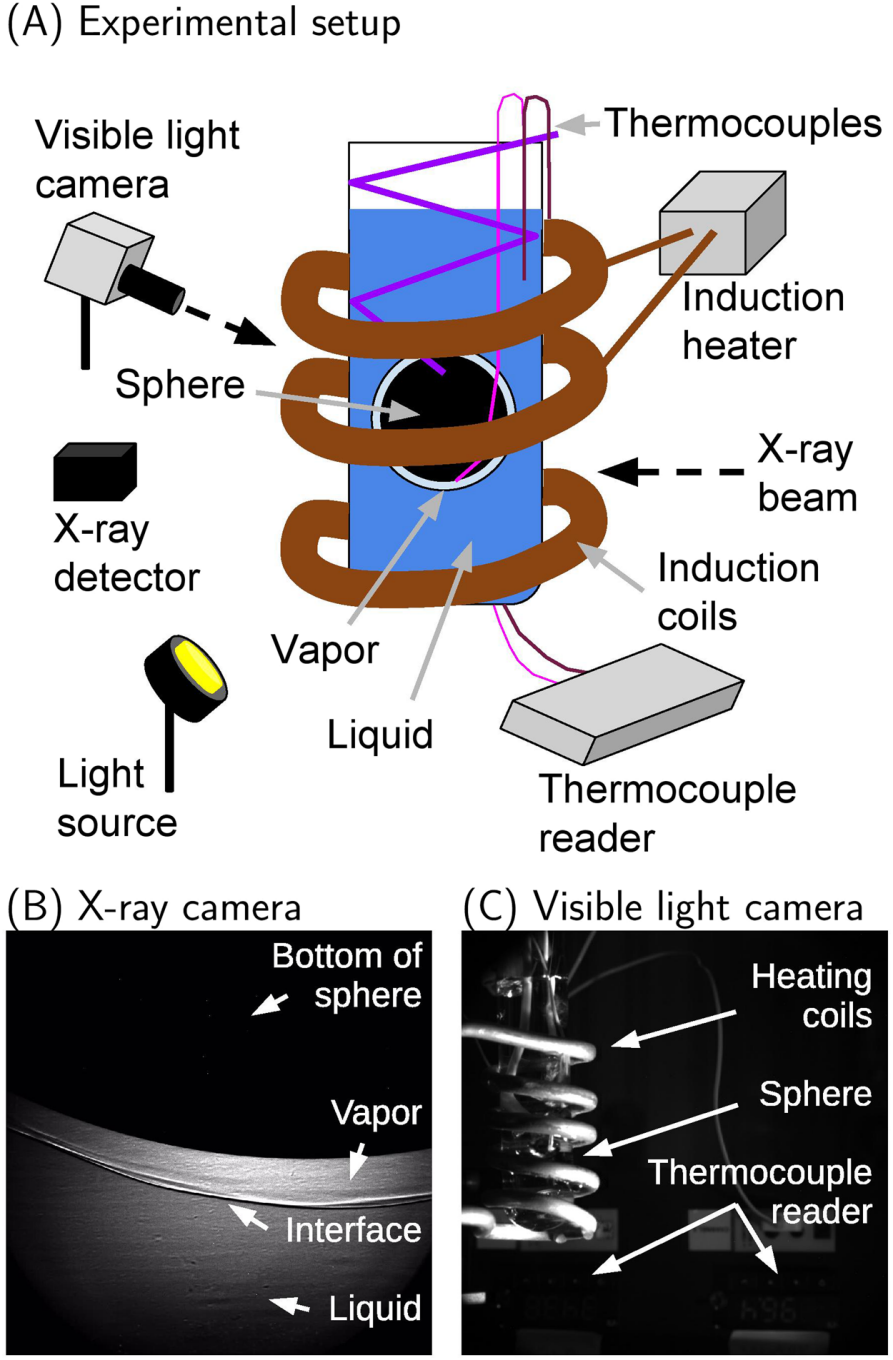
Phase-contrast imaging experiment. (**A**) Experimental setup at the Advanced Photon Source. (**B**) X-ray image of the liquid-vapor interface at the bottom of the heated sphere. The brightness and contrast of the X-ray image has been enhanced for clarity. (**C**) Visible light image corresponding to the X-ray image. The surface and water temperatures are located beneath the induction coils, and were read upside-down.

The bottom surface of the samples was imaged by the X-rays, as this location was not subject to frequent bubble departure like the top surface. X-ray images were synchronized with a second high-speed visible light camera (FASTCAM SA1.1 model 675K-M1) targeted at the entire sample, along with the thermocouple reader. The experimental setup is shown in Fig. 1. Figure 1A shows (in clock-wise direction), an X-ray beam, a thermocouple reader, light source, X-ray image detector, second high-speed visible light camera, and induction heater that encompassed the sample in the center.

The X-ray camera recorded images at 240 frames per second (fps) using a shutter speed of 1/15094 seconds. The visible light camera used a frame rate of 60 fps, and a shutter speed of 1/60 seconds. These frame rates were maintained throughout the experiments to ensure synchronization between the X-ray and visible light cameras, and more importantly to acquire images of the full formation and collapse process. Figure 1B shows the information obtained from the X-ray beam. The bottom of the sphere is shown at the top of the image, with an adjacent vapor film. A liquid-vapor interface separates the vapor film from the bulk liquid. Figure 1C shows the corresponding synchronized image from the visible light camera. The copper heating coils form a helix around the sample placed in a test tube filled with water. The location of two thermocouple readers are indicated. When the image is brightened, the displays yield temperature measurements of the sphere and liquid water, that are read upside-down.

## Results

### Experimental results

Four experimental trials are reported for the smooth sphere (SM), rough sphere (RO), and three trials for the thermoprobe (TP). These trials were designed to capture both the formation and collapse of the vapor film. This process required rapid heating to prevent the ambient water from completely boiling into vapor, but without permanently saturating the thermocouple reader by exceeding the maximum device temperature of 999.9 °C. This was a limitation from the geometry of the heating coil and test tube used. Thus, results from a few of the intermediate trials were not recorded.

#### Phase-contrast X-ray images of the film collapse process

**Figure 2:**
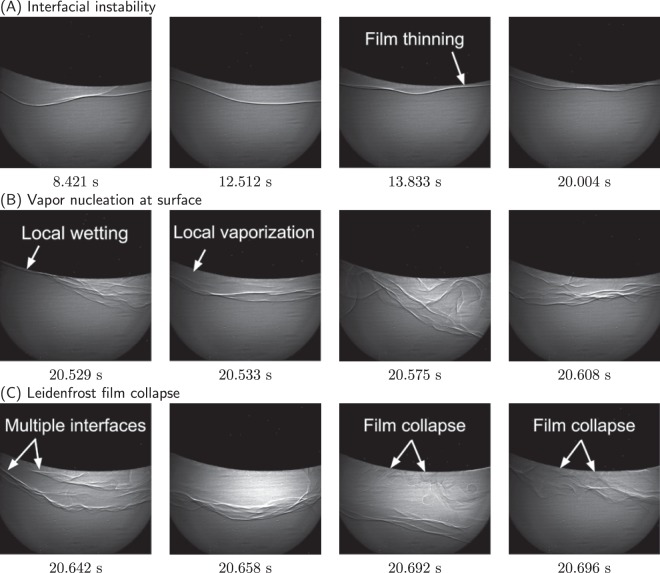
X-ray images of the Leidenfrost film collapse process for trial RO3 of the rough sphere. The brightness and contrast of each image has been enhanced for clarity. Image dimensions correspond to ~1914 *μm* × 1914 *μm*. (**A**) Interfacial instability. (**B**) Local wetting of the surface, followed by vapor nucleation and recovery of the liquid-vapor interface. (**C**) Leidenfrost film collapse.

The vapor film formed around each sample after several violent iterations of liquid contacting the surface, and subsequently vaporizing away in the form of bubbles with increasing size. The bubbles eventually coalesced into a single film that encompassed the samples. Upon formation of the film, external heat was no longer supplied to the samples. For each sample, the liquid-vapor interface fluctuated in both time and space as shown in Fig. 2A. The X-ray images in Fig. 2 were enhanced for clarity. See Fig. S4 for a comparison of the enhanced image to the original image.

Film thinning of the liquid-vapor interface was observed for all trials. This is shown in Fig. 2A where the liquid-vapor interface neared the surface. Prior to the final film collapse, liquid wet the surface (Fig. 2B), and then vaporized at the point of contact. This was evident by the disruption of the interface, along with the restoration of the film in subsequent X-ray images. Local contact of liquid with the solid sphere was non-terminal, but ranged in frequency across trials. This process of interfacial fluctuations, followed by local wetting and vaporization was cyclic until the liquid fully wet the surface during the final film collapse (Fig. 2C). Note: for trials SM2-SM3, the Leidenfrost collapse did not occur within the field of view. See Fig. S5 for X-ray images. Movies showing the entire film formation and collapse are provided in Supporting Movies S1–S11.

#### The Leidenfrost temperature and effect of surface roughness

**Figure 3:**
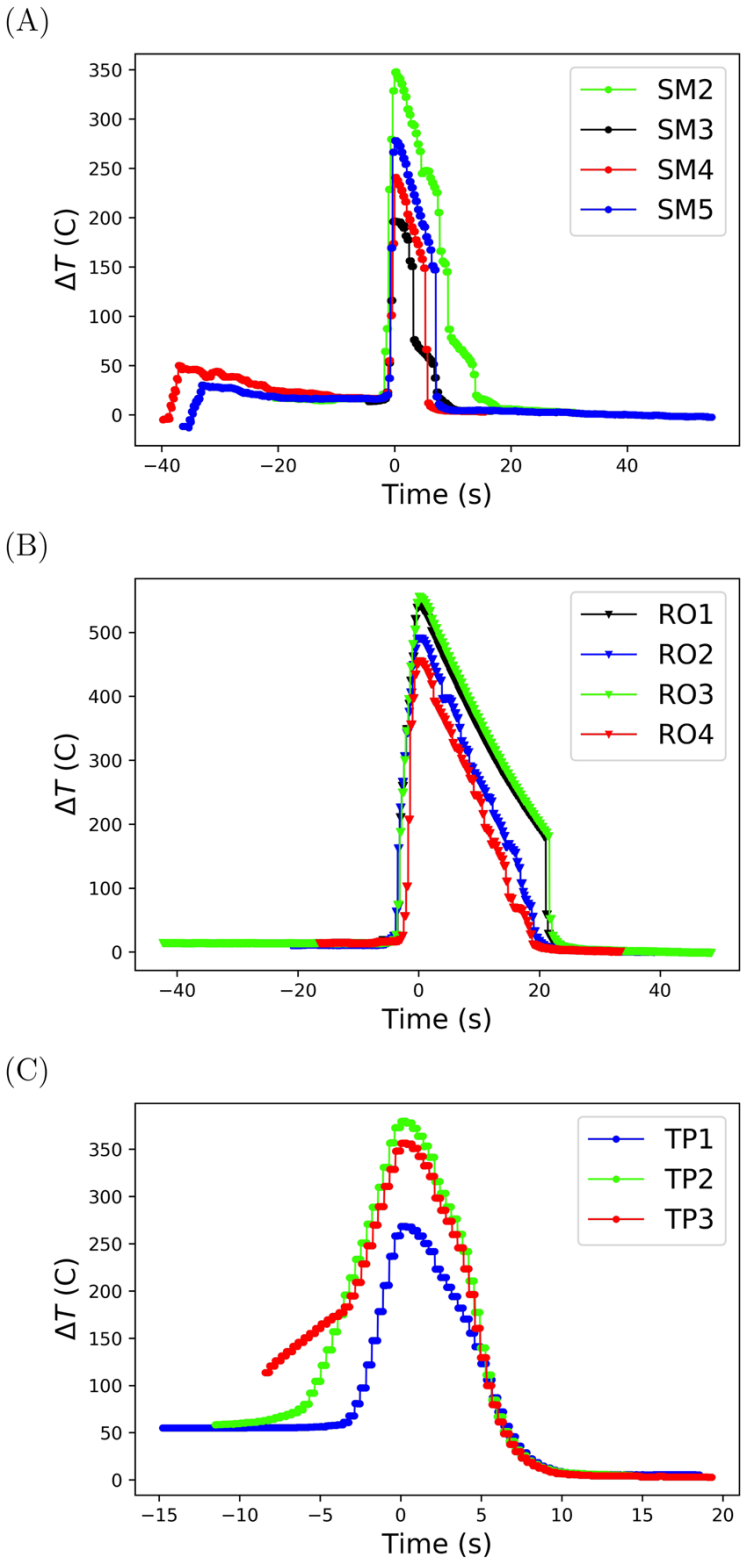
Wall superheat Δ*T* = *T*_*s*_ − *T*_*W*_ vs. cooling time. (**A**) Smooth sphere. (**B**) Rough sphere. (**C**) Thermoprobe. The data were linearly connected for visual aid.

The surface temperature *T*_*s*_ and bulk water temperature *T*_*w*_ were extracted from the thermocouple reader display in the visible light image (Fig. 1C). The superheat Δ*T* = *T*_*s*_ − *T*_*w*_ for each trial is plotted in Fig. 3 for both spheres and the thermoprobe. Trials SM2-SM3 of the smooth sphere demonstrated multiple sudden decreases in temperature. This was due to multiple contact between the liquid and the sphere. Trials SM4-SM5 exhibited the canonical abrupt decrease in temperature. On the rough sphere, trial RO1 and trial RO3 behaved as expected. Trial RO2 and trial RO4 did not experience drastic changes in temperature. Instead, a gradual decrease was observed until the superheat neared zero. Examining the X-ray images, frame-by-frame (Fig. S6), confirmed that liquid was continuously wetting and then dewetting the surface. This is consistent with experiments that measured changes in electrical impedance, which concluded liquid-solid contact to be present in both the transition and film boiling regimes^16–20^.

The measured Leidenfrost superheat Δ*T*_*LFP*_ are reported in Table 2. These values are not reported for trial RO2 and trial RO4, as a stable film was never achieved. The average Leidenfrost superheat (with standard deviation) was Δ*T*_*LFP*_ = 157.0 ± 5.7 °C for the smooth sphere, Δ*T*_*LFP*_ = 191.2 ± 0.4 °C for the rough sphere, and Δ*T*_*LFP*_ = 187 ± 13 °C for the thermoprobe. Surface roughness increased the temperature of the Leidenfrost film collapse. This finding is consistant with Leidenfrost temperatures found in literature^7–13^. In this case, applying sandpaper to the sphere surface was sufficient to elevate the Leidenfrost superheat by (191 °C–157 °C)/157 °C = 22%.

**Table 2:** Initial vapor film thickness.

Trial	${\langle {\delta }_{lv}^{min}\rangle }_{0}$ (*μm*)	${\langle {\delta }_{lv}^{mean}\rangle }_{0}$ (*μm*)	${\langle {\delta }_{lv}^{med}\rangle }_{0}$ (*μm*)	Δ*T*_*LFP*_ (°C)
SM2	109.75 ± 93.74	201.74 ± 86.33	201.03 ± 86.74	156
SM3	113.23 ± 84.95	200.26 ± 118.63	201.62 ± 121.22	156
SM4	54.68 ± 45.86	139.76 ± 63.41	141.33 ± 63.99	165
SM5	77.18 ± 68.06	191.91 ± 93.26	190.87 ± 95.87	151
RO1	153.76 ± 106.73	270.69 ± 154.53	265.99 ± 157.45	191
RO2*	207.62 ± 144.12	370.9 ± 226.29	371.12 ± 229.22	—
RO3	200.31 ± 167.28	312.1 ± 194.29	308.5 ± 198.68	191
RO4*	150.44 ± 72.61	223.31 ± 69.97	223.59 ± 68.7	—
TP1*	164.28 ± 82.56	312.38 ± 153.05	319.05 ± 161.91	106
TP2	285.01 ± 276.04	401.16 ± 252.73	415.71 ± 280.09	178
TP3	198.45 ± 126.07	273.87 ± 138.06	275.11 ± 139.99	196
〈SM〉	88.71 ± 27.9	183.42 ± 29.42	183.72 ± 28.68	157 ± 6
〈RO〉	177.03 ± 32.91	291.39 ± 29.28	287.24 ± 30.06	191 ± 0
〈TP〉	241.73 ± 61.21	337.51 ± 90.01	345.41 ± 99.42	187 ± 13

The minimum ${\langle {\delta }_{lv}^{min}\rangle }_{0}$, mean ${\langle {\delta }_{lv}^{mean}\rangle }_{0}$, and median ${\langle {\delta }_{lv}^{med}\rangle }_{0}$ film thickness, along with the Leidenfrost superheat Δ*T*_*LFP*_ are calculated. The standard deviation follows the “±” symbol. *Values are reported, but excluded from the analysis, as a stable film never formed (RO2, RO4) or a significant amount of water suddenly evacuated the test tube (TP1).

### Analysis of the vapor film thickness

The thickness and stability of the vapor film have implications for heat transfer from the surface to the liquid. To derive insight about the film collapse process, an algorithm was developed to parse the thousands of X-ray images per experimental trial. This algorithm obtained the dynamic vapor film thickness in both time and space. Multiple interfaces within the same X-ray frame were due to superposition, and were not differentiated by the algorithm. See Materials and Methods, and Figs S7 and S8 for details.

#### Film thickness calculations

**Figure 4:**
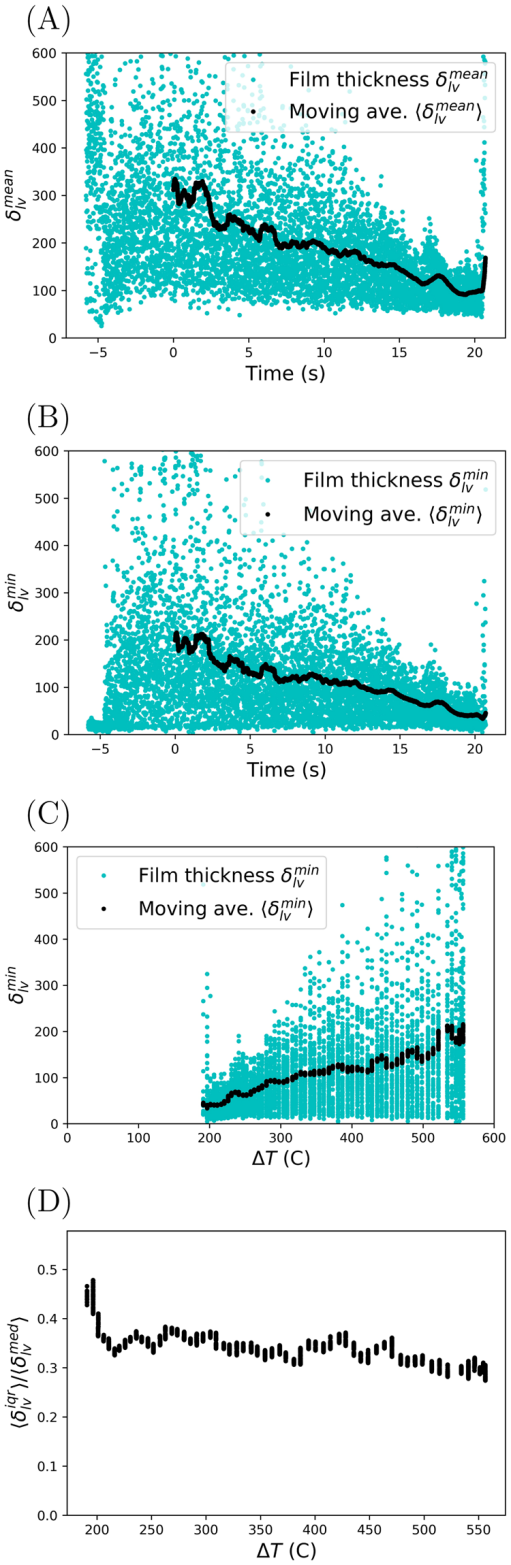
Measuring the vapor film thickness on a rough sphere (trial RO3). (**A**) Mean film thickness vs. cooling time. (**B**) Minimum film thickness vs. cooling time. (**C**) Minimum film thickness vs. superheat Δ*T* for cooling times *t* ≥ 0. (**D**) Interquartile range relative to the median film thickness vs. superheat Δ*T* for cooling times *t* ≥ 0.

The time-dependent mean and minimum film thickness for trial RO3 of the rough sphere are plotted in Fig. 4. A time-based moving average was plotted to illustrate the overall trend of the film thickness. The number of points averaged was determined by maximizing an effective signal-to-noise ratio ${\langle {\delta }_{lv}^{x}\rangle /\langle {\sigma }_{{\delta }_{lv}^{x}}\rangle |}_{t=0}$ during the time cooling began. Here, *x* = [*min*, *med*, *mean*] and $\langle {\sigma }_{{\delta }_{lv}^{x}}\rangle$ is the moving standard deviation. This improved film thickness estimates provided in Table 2, while still capturing the overall trend.

**Figure 5:**
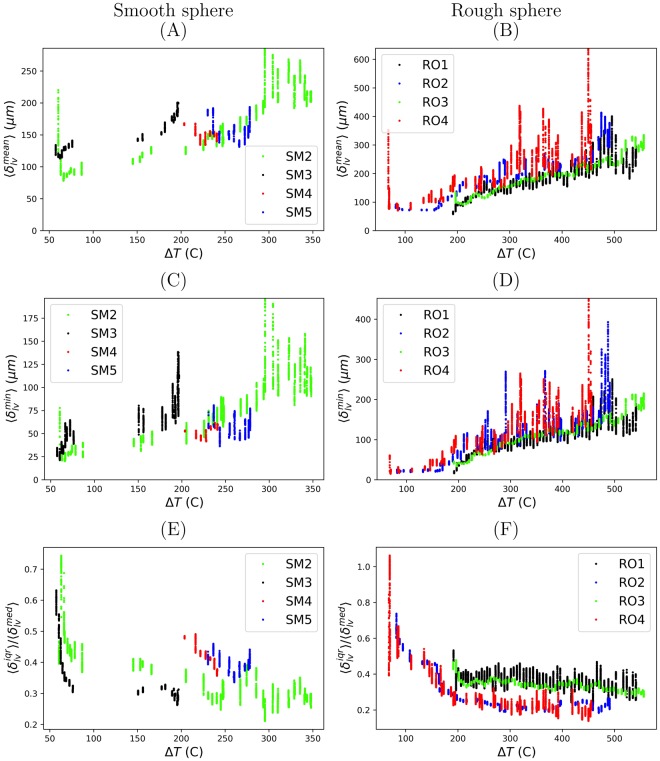
Moving averages of film thickness vs. superheat Δ*T* for cooling times *t* ≥ 0. (**A**,**B**) Average mean vapor film thickness $\langle {\delta }_{lv}^{mean}\rangle$. (**C**,**D**) Average minimum vapor film thickness $\langle {\delta }_{lv}^{min}\rangle$. (**E**,**F**) Interquartile range relative to the median film thickness.

For trial RO3, the average mean film thickness when cooling began was ${\langle {\delta }_{lv}^{mean}\rangle }_{0}=312.1\pm 194.29\,\mu m$, whereas, the average minimum film thickness was ${\langle {\delta }_{lv}^{{\min }}\rangle }_{0}=200.31\pm 167.28\,\mu m$. In Fig. 4A–C, the collapse of the Leidenfrost film is observed when the moving average reached a minimum value. The film thickness (${\delta }_{lv}^{min}$, ${\delta }_{lv}^{mean}$, and ${\delta }_{lv}^{med}$) were measured for each trial of the smooth and rough spheres. To reduce the amount of data reported into accessible plots, moving averages of the minimum $\langle {\delta }_{lv}^{min}\rangle$, mean $\langle {\delta }_{lv}^{mean}\rangle$, and interquartile range of the film thickness are plotted in Fig. 5 as a function of superheat and Figs S9 and S10 as a function of time. For the rough sphere, moving averages of the mean $\langle {\delta }_{lv}^{mean}\rangle$ and minimum $\langle {\delta }_{lv}^{min}\rangle$ film thickness decreased with decreasing superheat for each trial. This was also the case for trials SM2-SM3 of the smooth sphere. For SM2-SM3, there is a gap between ${\rm{\Delta }}T\in [87,145]$ °C because the film collapsed outside the field of view of the X-ray camera (i.e. in a region we were not looking).

Variability between trials was due to corrosion of the stainless steel spheres caused by the induction heating while underwater. This was evident by the blackened colored surface finish. This resulted in the need to increase the initial supply power in response to the degradation in heat transfer from the spheres to the water caused by corrosion. Additionally, local surface defects also influenced the results – particularly for the roughened surface.

#### Spatial fluctuations

The film thickness experienced large spatial variation throughout the collapse process. To quantify the variability, the interquartile range of the film thickness $\langle {\delta }_{lv}^{iqr}\rangle$ relative to the median film thickness $\langle {\delta }_{lv}^{med}\rangle$ was plotted in Fig. 4D for RO3 and Fig. 5E,F for the other trials. The interquartile range represents the spread of film thicknesses between the 25^*th*^ percentile and the 75^*th*^ percentile. The ratio $\langle {\delta }_{lv}^{iqr}\rangle /\langle {\delta }_{lv}^{med}\rangle$ quantifies the spread of film thicknesses relative to the second quartile $\langle {\delta }_{lv}^{med}\rangle$, for which the interquartile range is centered. For $\langle {\delta }_{lv}^{iqr}\rangle /\langle {\delta }_{lv}^{med}\rangle =2.0$, the film thickness is of the same magnitude as the variation about $\langle {\delta }_{lv}^{med}\rangle$ and the lower 25^*th*^ percentile will be touching the surface. For $\langle {\delta }_{lv}^{iqr}\rangle /\langle {\delta }_{lv}^{med}\rangle =0.5$, half of the film thickness measurements will be between the range $\mathrm{(3}/\mathrm{4)}\times \langle {\delta }_{lv}^{med}\rangle$ to $\mathrm{(5}/\mathrm{4)}\times \langle {\delta }_{lv}^{med}\rangle$. This means 25% of the film thickness measurements reside below $\mathrm{(3}/\mathrm{4)}\times \langle {\delta }_{lv}^{med}\rangle$. Lastly, for $\langle {\delta }_{lv}^{iqr}\rangle /\langle {\delta }_{lv}^{med}\rangle =0$, there is no variation in the film thickness. In Fig. 4D, $\langle {\delta }_{lv}^{iqr}\rangle /\langle {\delta }_{lv}^{med}\rangle$ increased from a minimum of 0.27 to a maximum of 0.48 prior to film collapse. This suggests the distribution of fluctuations, in particular the lower quartile, also influences the collapse process.

#### Discrete fast Fourier transform

**Figure 6:**
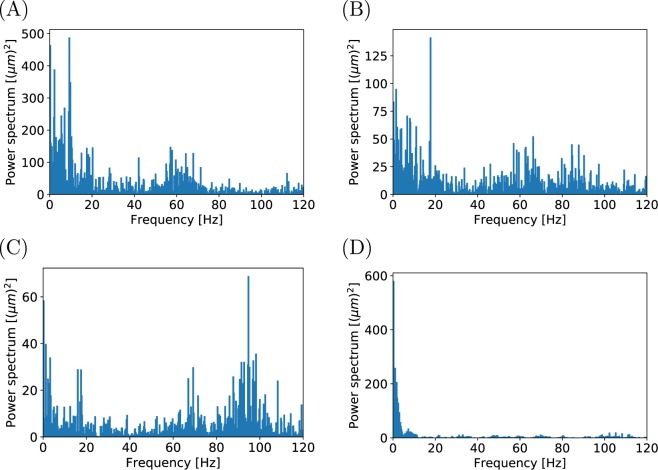
Discrete fast Fourier transform of the mean film thickness $({\delta }_{lv}^{mean}-mean\,[{\delta }_{lv}^{mean}])$ for trial RO3 of the rough sphere. (**A**) Beginning of cooling period 0.0–4.0 s. (**B**) Middle of cooling period 5.18–9.18 s. (**C**) Middle of cooling period 10.35–14.35 s. (**D**) Period before film collapse 16.71–20.71 s.

In addition to spatial fluctuations, the film thickness also varied with time. To quantify these fluctuations, a discrete fast Fourier transform was implemented across four periods, lasting four seconds each, to observe the frequency response before the film collapsed. This is shown in Fig. 6 for trial RO3 of the rough sphere.

The transform was computed on the difference between the mean film thickness ${\delta }_{lv}^{mean}$ and the average film thickness $mean\,[{\delta }_{lv}^{mean}]$ over the four second period. This was done to focus on the fluctuations, and not the zero frequency component of the spectrum. Since the X-ray camera obtained measurements at 240 Hz, frequencies above the Nyquist frequency were aliased to below 120 Hz.

For trial RO3, the four equivalently spaced periods shown in Fig. 6 were 0.0–4.0 s, 5.18–9.18 s, 10.35–14.35 s, and 16.71–20.71 s. The dominant non-zero frequency for each period was 9.26 Hz, 17.75 Hz, 94.75 Hz, and 0.25 Hz. For comparison, we repeated the discrete fast Fourier transform on trial RO4 across the four second periods: 0.0–4.0 s, 4.18–8.18 s, 8.36–12.36 s, and 12.73–16.73 s. This is shown in Fig. S11. The dominant non-zero frequency for each period of trial RO4 was 0.25 Hz, 0.5 Hz, 0.5 Hz, and 0.25 Hz.

#### Frequency of liquid-solid contact

Liquid water repeatedly touched the heated surfaces throughout the film boiling process. To quantify the frequency of liquid-solid contact, the duration between sequential liquid-solid contact events was inverted. Contact was defined to occur when the resolution of the minimum film thickness ${\delta }_{lv}^{min}$ was less than or equal to eight pixels. This resolution corresponded to half the width of the liquid-vapor interface and a physical resolution of ~14.8 *μm* for the smooth sphere, ~15.2 *μm* for the rough sphere, and ~28.5 *μm* for the thermoprobe, depending upon the moving position of the sample. See Fig. S12 for calculated frequencies.

Unfortunately, the frame rate of the X-ray camera was too low to observe all of the contact events. This was confirmed with separate X-ray tests on larger spheres sampled at 2000 Hz, in which the liquid-vapor interface still moved sufficiently. Despite this, the under-sampled frequencies reported in Fig. S12 still show that liquid-solid contact is occurring throughout the film collapse process.

## Discussion

The Leidenfrost collapse process can be understood as a competition between mechanisms that destabilize the vapor film and mechanisms that stabilize the vapor film. Interfacial instabilities, which may be driven by differences in temperature, density, surface energy, shear flow, or phase-change heat transfer, are destabilizing. By contrast, vapor formation near the surface serves to stabilize the film. Vapor formation is influenced by the surface wettability, surface heat transfer, nucleation site density, among others.

The experiments reported in this study observe the film collapse process, which is inherently non-equilibrium. This non-equilibrium process encompasses both slow and fast timescales. Cooling of the sphere is a slow process. For trial RO3, the sphere reached thermal equilibrium after ~20 *s* of natural cooling. Fluctuations in the film thickness about a mean value occur on a fast timescale ($\ll 1\,s$) until contact with the sphere. Once contact is made, and a stabilizing vapor has nucleated, the film thickness will fluctuate about a new mean thickness.

One strength of the X-ray imaging technique is that the absolute film thickness and liquid-solid contact can be directly observed, which cannot be achieved by counting fringes in light interference patterns^21,22^. X-ray images (Fig. 2, Supporting Movies S1–S11) of the liquid-vapor interface confirm an interfacial instability to be present during the film collapse process. This instability is not considered to be the Rayleigh-Taylor interfacial instability, as it occurred on the bottom surface of the samples, where the less-dense vapor resided above the denser liquid water. Fluctuations were quantified by temporal measurement of (1) the absolute vapor film thickness, (2) the interquartile range relative to the median film thickness, and (3) a discrete fast Fourier transform of the mean film thickness. The interquartile range was more representative of the spatial variation than the difference between the maximum and minimum film thickness. The same X-ray images (Fig. 2) also confirm liquid contacting the heated surface, and then creating a vapor layer throughout the cooling process. This occurred until fluctuations in the liquid-vapor interface overwhelmed the formation of vapor, leading to the final film collapse. From this, we conclude the nucleation of vapor to be a second mechanism present during the film collapse process.

The minimum film thickness $\langle {\delta }_{lv}^{min}\rangle$ was informative for predicting when the Leidenfrost film would collapse. This is shown in Fig. 5D for each trial of the rough sphere. Additionally, the slope of the minimum film thickness with respect to the superheat may further explain the behavior of trial RO2 and trial RO4 of the rough sphere (Fig. 5D). These trials both experienced gradual decreases in the film thickness, which correspond to the continuous decreases observed in the superheat (Fig. 3B) prior to film collapse. This was not the case for trial RO1 and trial RO3, for which the film rapidly collapsed.

The boiling experiments were limited by (1) the measurement of temperature, and (2) the X-ray field of view. Two K-type thermocouples, that were outside the X-ray field of view, were attached to the spheres. The larger-gauge thermocouple was used to suspend the sphere within water, whereas, the smaller-gauge thermocouple measured the sphere temperature. We desired the smaller-gauge thermocouple to reduce the presence of the wires. Based on observations of the visible light camera and X-ray camera, it did not appear the thermocouples influenced the film thickness results. Additionally, the vapor film thickness became smaller than the size of the thermocouples, which indicates the film did not collapse due to the presence of the thermocouples. An additional small-gauge K-type thermocouple was suspended in the bulk water to measure the ambient liquid temperature. The thermocouple data was used to deduce the Leidenfrost temperature. The two small-gauge thermocouples were connected to a thermocouple reader with limited response time. This lowered the resolution of temperature measurements.

The X-ray imaging technique, and induction heater selection were both responsible for the limited field of view. The maximum power supplied by the induction heater to the stainless steel spheres was dictated by the heater, as well as the fixed geometry of the helical induction coils. This placed a constraint on the sphere diameter, as smaller spheres required greater power to achieve film boiling. A stronger heater would have permitted an entire small-sized sphere to undergo X-ray imaging. However, it was not feasible for us to acquire a stronger heater solely for this experiment. Instead, we focused our efforts on a region of interest that we could image – the bottom of the sphere.

The liquid-vapor interface at the bottom of the sphere maintained a persistent interface once a vapor film was formed. The vapor film was not necessarily stable, but the interface was well-defined and observable, leading to useful results. We were interested in film collapse events that occurred on the bottom surface of the sphere, where the direction of the gravitational force stabilizes the vapor film. For several experimental trials (trials RO1-RO4, SM4-SM5, TP1-TP3), observations of the bottom surface directly corresponded to film collapse events. For other cases (trials SM2-SM3), they did not. Had we imaged the left or right sides of the samples, we would have seen interfaces influenced by vapor flow to the top of the samples. We initially looked at the top of the spheres, but were unable to image the liquid-vapor interface as bubbles were released throughout the heating/cooling process. The bubbles were larger than the vapor film thickness and distorted the liquid-vapor interface prior to rupture. This would have forced us to study bubble departure, as opposed to film thickness, which was not the intent of this study.

## Summary

This work has confirmed two of the mechanisms hypothesized to influence the Leidenfrost collapse. Mechanism 1: the interfacial instability does affect how liquid wets the surface, but only up until the point of contact with the surface. Once contact is made, mechanism 2: vaporization of the liquid determines whether the liquid-vapor interface will be recovered. This process of a destabilizing interfacial instability, followed by liquid contact, and then stabilizing vaporization is cyclic throughout the film boiling regime until the final film collapse.

The direct influence of other mechanisms, such as temperature-dependent wettability, or the influence of the liquid spinodal limit need further investigation. Future work will aim to repeat the Leidenfrost collapse experiments conducted at the Advanced Photon Source using a faster digital temperature acquisition system.

## Methods

### Algorithm for measuring the vapor film thickness

Phase-contrast X-ray images of the liquid-vapor interface were obtained for multiple trials during the experiment. To quantify these results, individual images were analyzed to obtain spatial and temporal coordinates of both the liquid-vapor interface and the sphere. Refer to Fig. 4 for the results of the algorithm applied to trial RO3 of the rough sphere. The algorithm, written in Python 3.6 using the OpenCV library, proceeded in generalized steps as follows:

**Step 1** Load TIFF image (1024 × 1024 pixel resolution)

**Step 2** Median filter with a 5 × 5 kernel to reduce image noise

**Step 3** Apply an adaptive Gaussian threshold to identify interfaces

**Step 4** Median filter three additional times with a 7 × 7 kernel to reduce image noise

**Step 5** Identify the coordinates of the sphere surface (*x*_*s*_, *y*_*s*_) and the liquid-vapor interface (*x*_*lv*_, *y*_*lv*_)

**Step 6** Fit a circle to the coordinates of the sphere surface (*x*_*s*_, *y*_*s*_) to obtain the radius *r*_*s*_ and center (*x*_*c*_, *y*_*c*_) of the sphere measured in pixels

The difference between the coordinates of the liquid-vapor interface (*x*_*lv*_, *y*_*lv*_) and the center coordinates of the sphere (*x*_*c*_, *y*_*c*_) yield a relative position

1 $${r}_{lv}(pix)=\sqrt{{({x}_{lv}-{x}_{c})}^{2}+{({y}_{lv}-{y}_{c})}^{2}}.$$

Subtracting the radius of the sphere *r*_*s*_(*pix*) from the relative position of the interface yields the vapor film thickness

2 $${\delta }_{lv}(pix)={r}_{lv}(pix)-{r}_{s}(pix).$$

When the film thickness was larger than the field of view (unidentified liquid-vapor interface), the film thickness was prescribed to be the field of view of the X-ray camera. While this did not occur often, it was necessary for representative moving averages. Measurements of the film thickness in pixels were converted to physical units by multiplying by the ratio of the experimental sphere radius *r*_*s*_(*μm*) to the sphere radius obtained from the image *r*_*s*_(*pix*) to achieve:

3 $${\delta }_{lv}(\mu m)={\delta }_{lv}(pix)\frac{{r}_{s}(\mu m)}{{r}_{s}(pix)}.$$

For a single image, the minimum, median, mean, and interquartile range of the film thickness are defined as:

4 $${\delta }_{lv}^{{\min }}(\mu m)=\,{\min }\,[{\delta }_{lv}(\mu m)],$$

5 $${\delta }_{lv}^{med}(\mu m)=median\,[{\delta }_{lv}(\mu m)],$$

6 $${\delta }_{lv}^{mean}(\mu m)=\overline{{\delta }_{lv}}(\mu m),$$

7 $${\delta }_{lv}^{iqr}(\mu m)=IQR\,[{\delta }_{lv}(\mu m)].$$

For ${\delta }_{lv}^{mean}$, outlier data was excluded based on a two standard deviation criterion to improve the fidelity of our measurements. The standard deviation is expected to be large, as the liquid-vapor interface fluctuated wildly throughout the film collapse process. See Fig. S7 for an example of the algorithm identifying the liquid-vapor interface. The moving averages (*mov*) and moving standard deviation (*movstd*) of the film thickness are defined as:

8 $$\langle {\delta }_{lv}^{{\min }}\rangle (\mu m)=mov\,[{\delta }_{lv}^{{\min }}(\mu m)],$$

9 $$\langle {\delta }_{lv}^{med}\rangle (\mu m)=mov\,[{\delta }_{lv}^{med}(\mu m)],$$

10 $$\langle {\delta }_{lv}^{mean}\rangle (\mu m)=mov\,[{\delta }_{lv}^{mean}(\mu m)],$$

11 $$\langle {\delta }_{lv}^{iqr}\rangle (\mu m)=mov\,[{\delta }_{lv}^{iqr}(\mu m)],$$

12 $$\langle {\sigma }_{{\delta }_{lv}^{x}}\rangle (\mu m)=movstd\,[{\delta }_{lv}^{x}(\mu m)].$$

The number of points averaged for each $\langle {\delta }_{lv}^{x}\rangle$, where *x* = [*min*, *med*, *mean*, *iqr*] was individually determined by maximizing an effective signal-to-noise ratio ${\langle {\delta }_{lv}^{x}\rangle /\langle {\sigma }_{{\delta }_{lv}^{x}}\rangle |}_{t=0}$ during the time cooling began. This was done to improve the film thickness values provided in Table 2. The number of points averaged for $\langle {\sigma }_{{\delta }_{lv}^{x}}\rangle$ was the same as the corresponding film thickness $\langle {\delta }_{lv}^{x}\rangle$.

## Electronic supplementary material

Supplementary Information

Supporting Movie S1

Supporting Movie S2

Supporting Movie S3

Supporting Movie S4

Supporting Movie S5

Supporting Movie S6

Supporting Movie S7

Supporting Movie S8

Supporting Movie S9

Supporting Movie S10

Supporting Movie S11

## Data Availability

Data generated or analyzed during this study are included in this published article, Supporting Information files, and the figshare public repository. Original TIFF images for each experiment are available from the corresponding author on reasonable request.
