# Supplementary material for: High-speed X-ray imaging of the Leidenfrost collapse
Source: Sci Rep. 2019 Feb 7;9:1598. doi: 10.1038/s41598-018-36603-w (PMC6367412; doi:10.1038/s41598-018-36603-w)
Supplement: Supplementary file 1 — Supplementary Information [file 41598_2018_36603_MOESM1_ESM.pdf]

Supporting Information: High-speed X-ray imaging of the Leidenfrost collapse

Paul R. Jones[1], **Chihpin (Andrew) Chuang**[2,3], **Tao Sun**[3], **Tom Y. Zhao**[1], **Kamel Fezzaa**[3], **Juan C. Takase**[1], **Dileep Singh**[2], **Neelesh A. Patankar**[1]

[1] Department of Mechanical Engineering, Northwestern University, 2145 Sheridan Road, Evanston, IL 60208, United States
[2] Argonne National Laboratory, Lemont, IL 60439, United States
[3] Advanced Photon Source, Argonne National Laboratory, Lemont, IL 60439, United States

* To whom correspondence should be addressed. E-mail: n-patankar@northwestern.edu

Supporting images

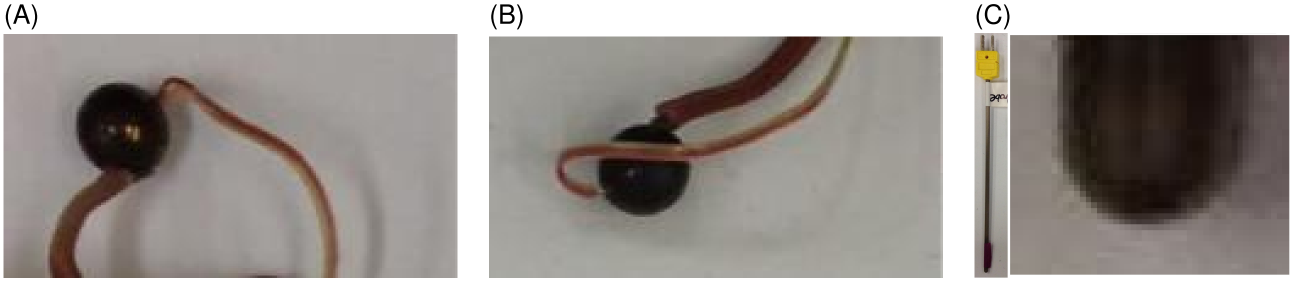

Figure S1. Samples used during imaging experiments. (A) Smooth sphere. (B) Rough sphere. (C) Thermoprobe. Two thermocouples were spot-welded to each sphere. The samples darkened in color during each trial of the experiment.

Jones, Paul R to me 12:00 AM

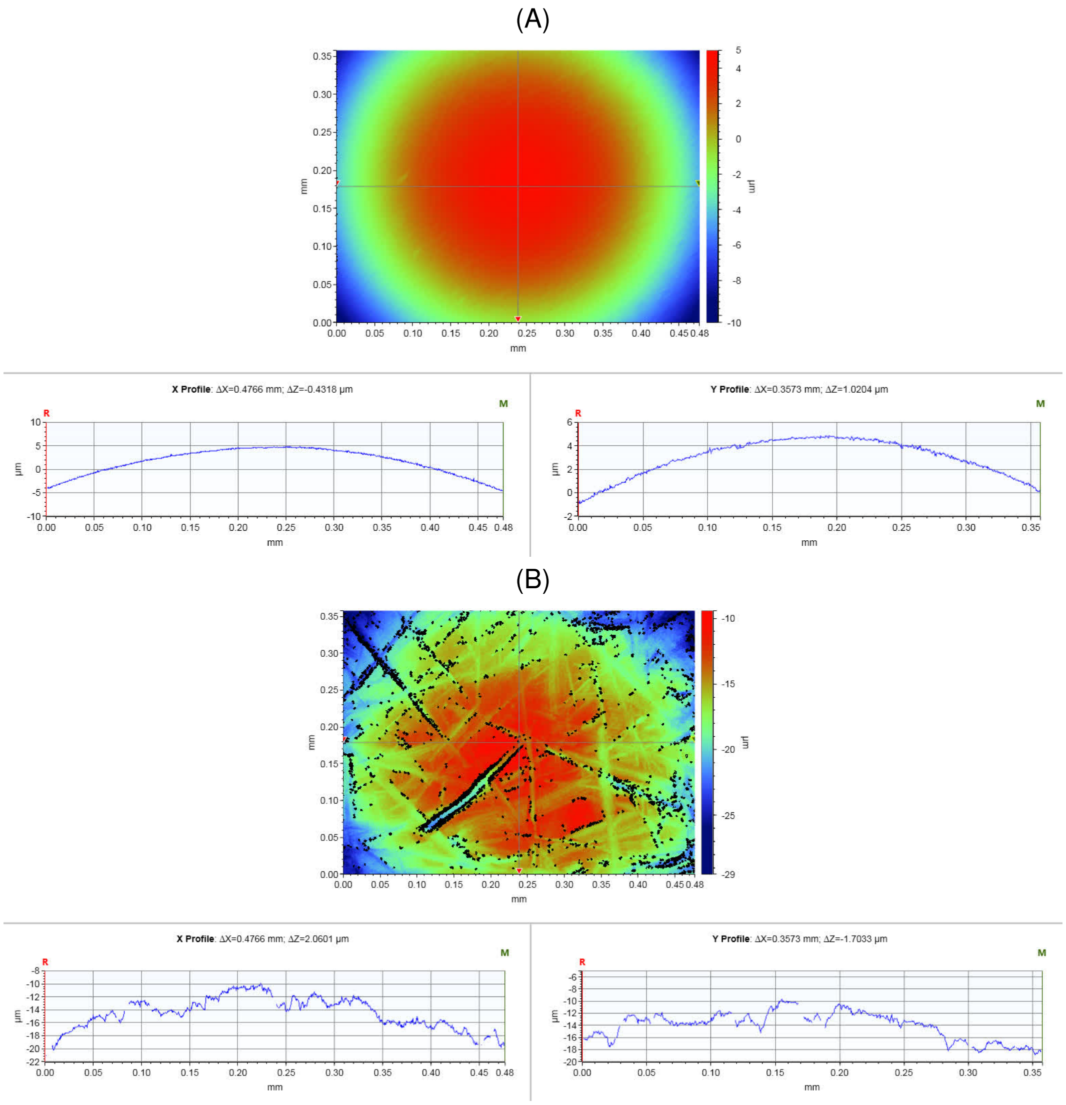

Figure S2. Profilometer characterization of spheres. Images obtained with 10x lens magnification. (A) Smooth sphere. (B) Rough sphere.

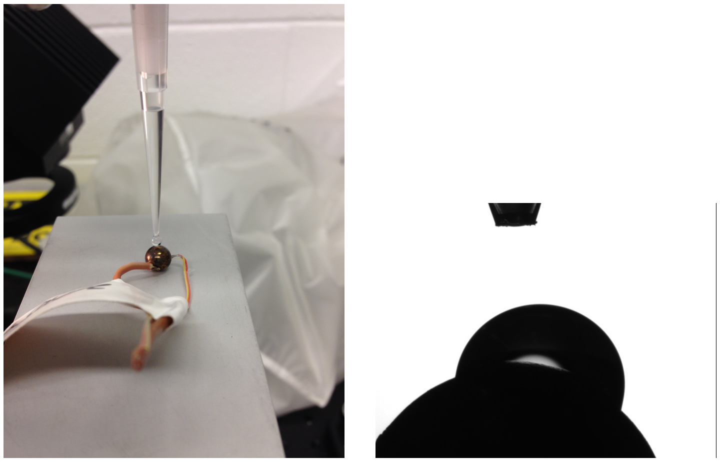

Figure S3. Contact angle measurement. Water droplet (10 μL) on the rough sphere using a goniometer. Contact angles θ post-experiment were estimated using MATLAB. Smooth sphere $\theta \sim 46.6 \pm 5.1°$. Rough sphere $\theta \sim 61.3 \pm 6.6°$.

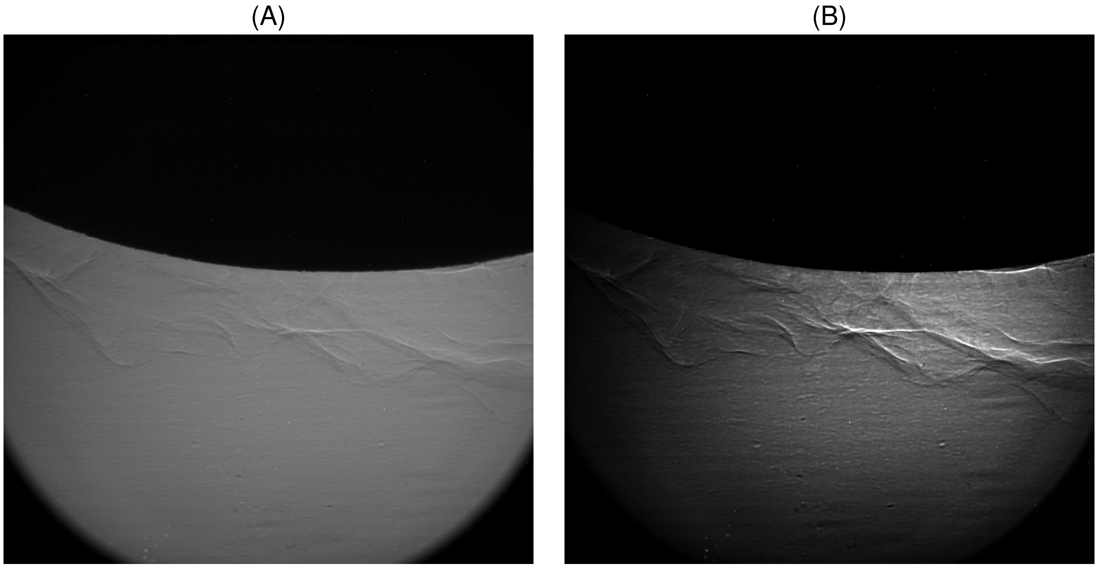

Figure S4. Enhancement of X-ray images. Images correspond to trial RO3 of the rough sphere at 20.696 s of cooling. (A) Original image. (B) Image enhanced with increased brightness and contrast.

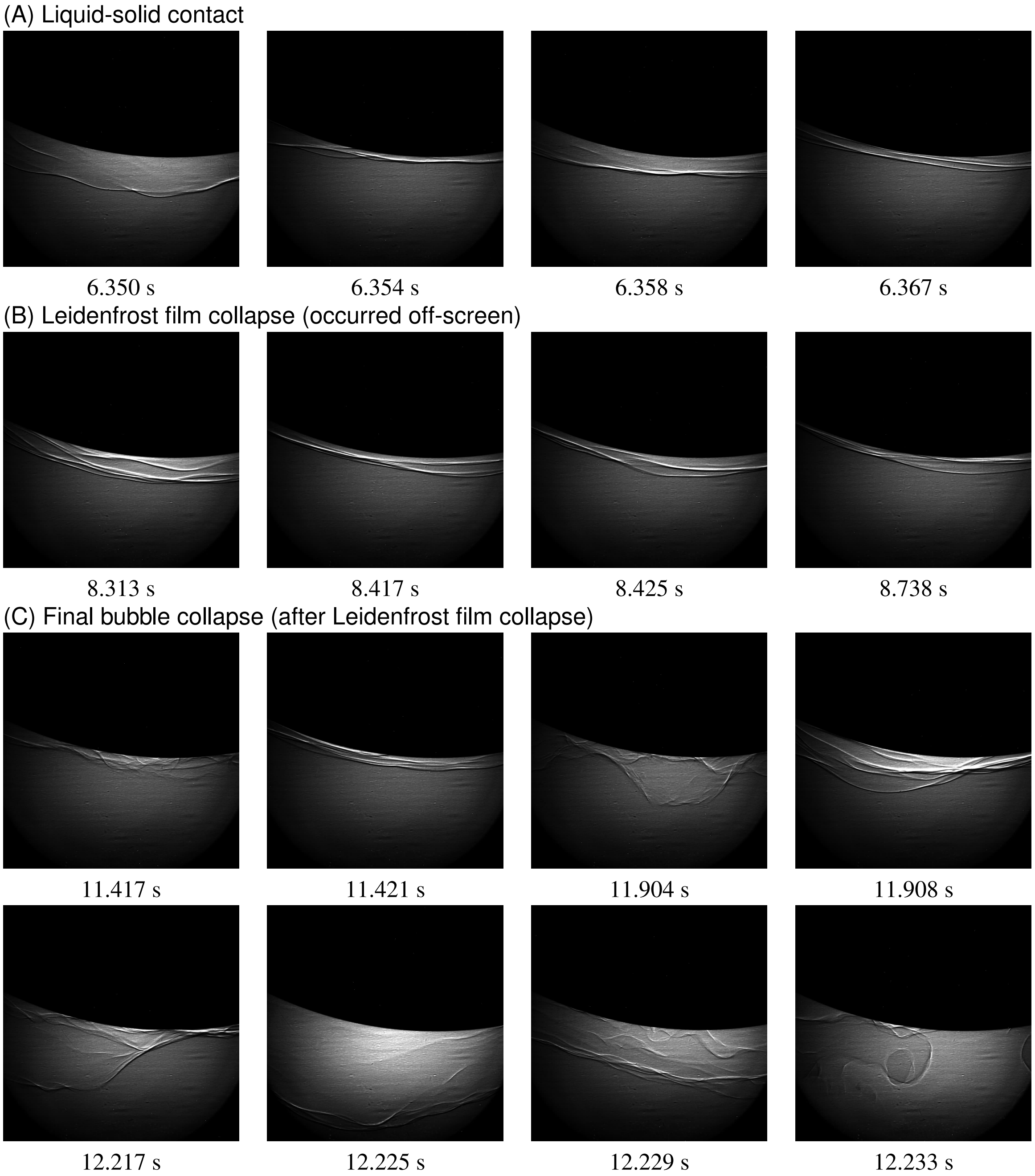

Figure S5. X-ray images of the Leidenfrost film collapse process (trial SM2). The brightness and contrast of each image has been enhanced for clarity. All images correspond to trial SM2 of the smooth sphere. The cooling time is provided below each image.

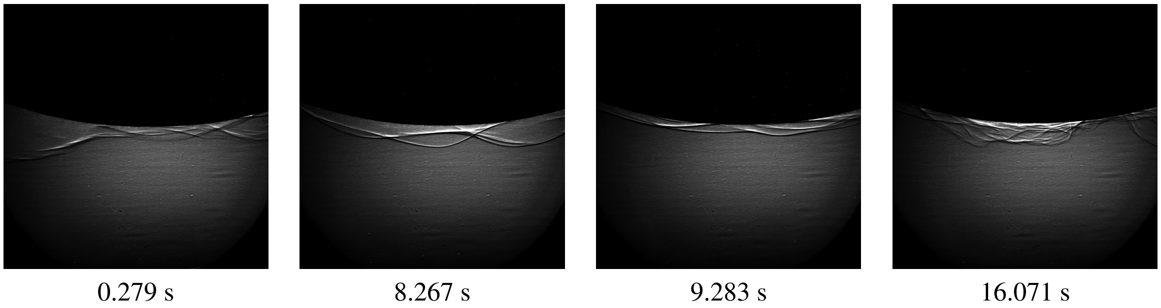

Figure S6. X-ray images of liquid-solid contact during the film collapse process (trial RO4). The brightness and contrast of each image has been enhanced for clarity. All images correspond to trial RO4 of the rough sphere. The cooling time is provided below each image.

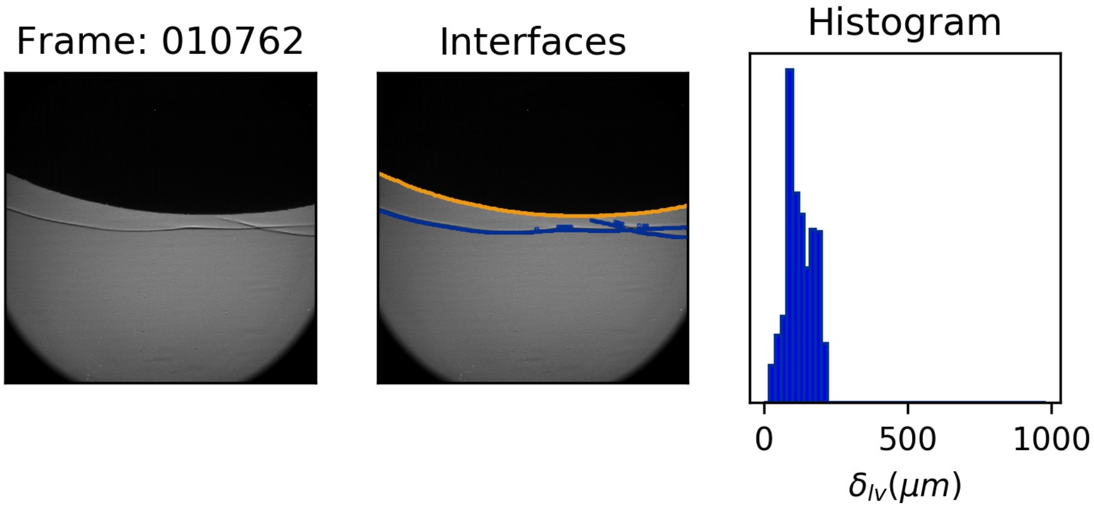

Figure S7. Measuring the vapor film thickness on a rough sphere (trial RO3). (A) X-ray image frame 10762. (B) Algorithm identification of the liquid-vapor interface and sphere surface. (C) Distribution of film thickness δ_{lv} for frame 10762.

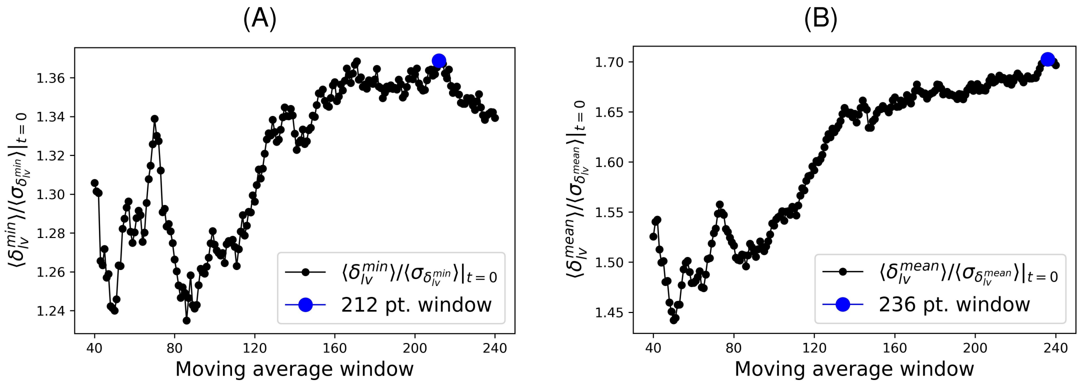

Figure S8. Optimal window size for moving averages of trial R3 The number of points averaged for each film thickness$\left(\langle\delta_{lv}^{min}\rangle, \langle\delta_{lv}^{mean}\rangle\right)$ was individually determined by maximizing an effective signal-to-noise ratio $\langle\delta_{lv}^{x}\rangle/\langle\sigma_{\delta_{lv}^{x}}\rangle|_{t=0}$, where $x = [min, mean]$ during the time cooling began. (A) Optimal window size for moving average of minimum film thickness. (B) Optimal window size for moving average of mean film thickness.

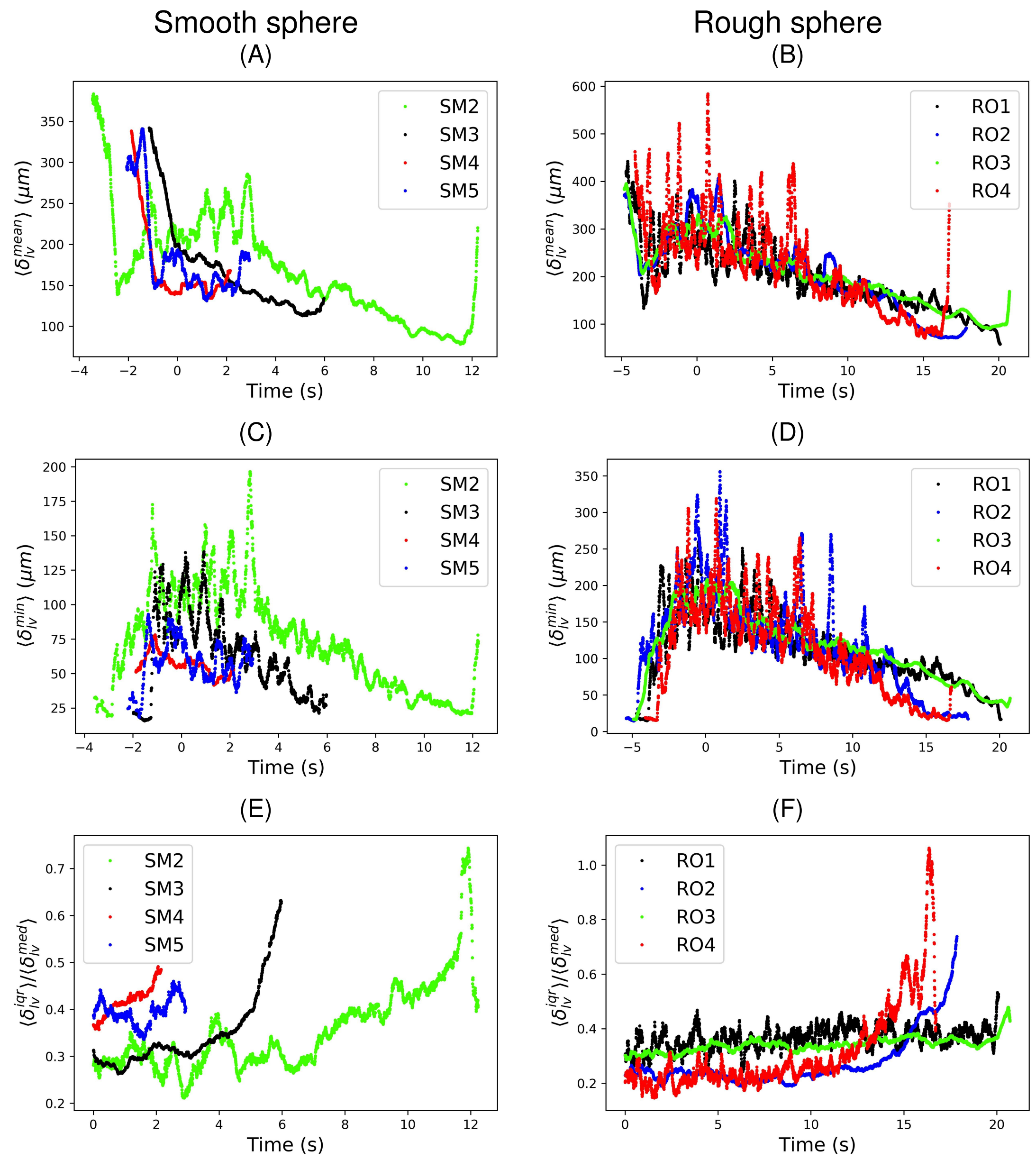

Figure S9. Moving averages of film thickness measurements on smooth and rough spheres. (A, B) Average mean vapor film thickness $\langle \delta_{lv}^{mean} \rangle$ vs. cooling time. (C, D) Average minimum vapor film thickness $\langle \delta_{lv}^{min} \rangle$ vs. cooling time. (E, F) Interquartile range relative to the median film thickness vs. cooling time.

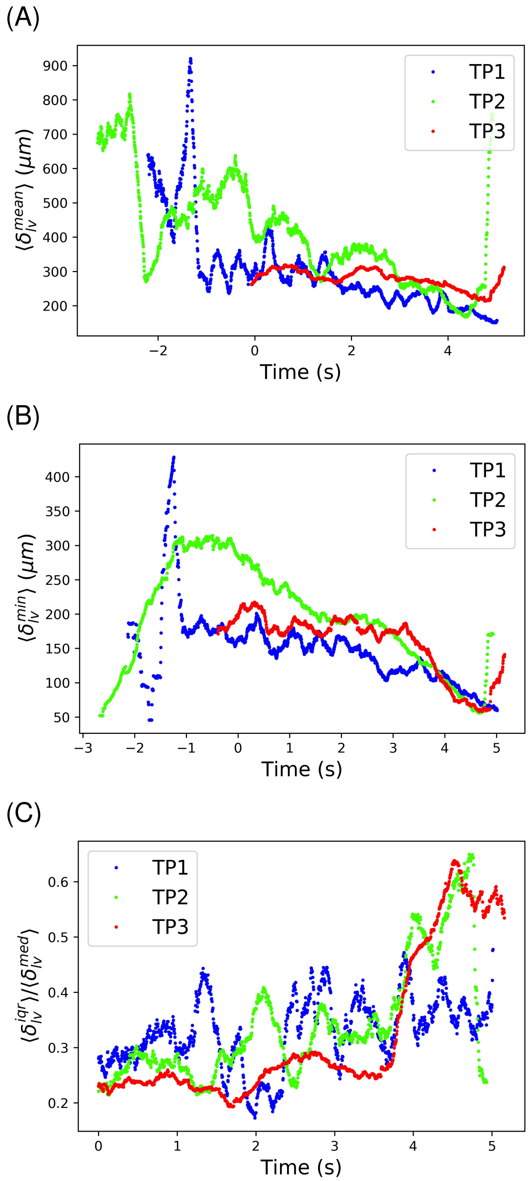

Figure S10. Moving averages of film thickness measurements on the thermoprobe. (A) Average mean vapor film thickness $\langle \delta_{lv}^{mean} \rangle$ vs. cooling time. (B) Average minimum vapor film thickness $\langle \delta_{lv}^{min} \rangle$ vs. cooling time. (C) Interquartile range relative to the median film thickness vs. cooling time.

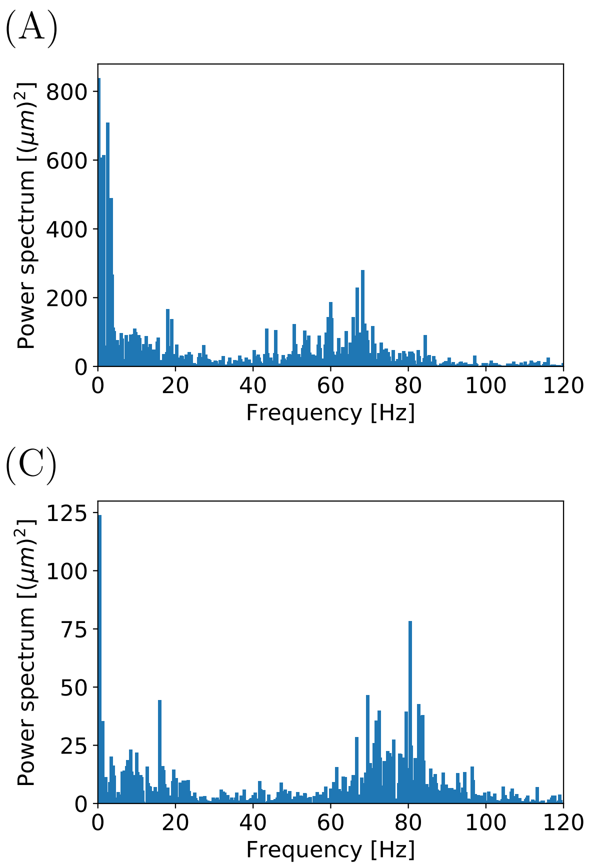

Figure S11. Discrete fast Fourier transform of the mean film thickness $\left(\delta_{lv}^{mean} - mean[\delta_{lv}^{mean}]\right)$ **for trial RO4 of the rough sphere.** (A) Beginning of cooling period 0.0-4.0 s. (B) Middle of cooling period 4.18-8.18 s. (C) Middle of cooling period 8.36-12.36 s. (D) Period before film collapse 12.73-16.73 s.

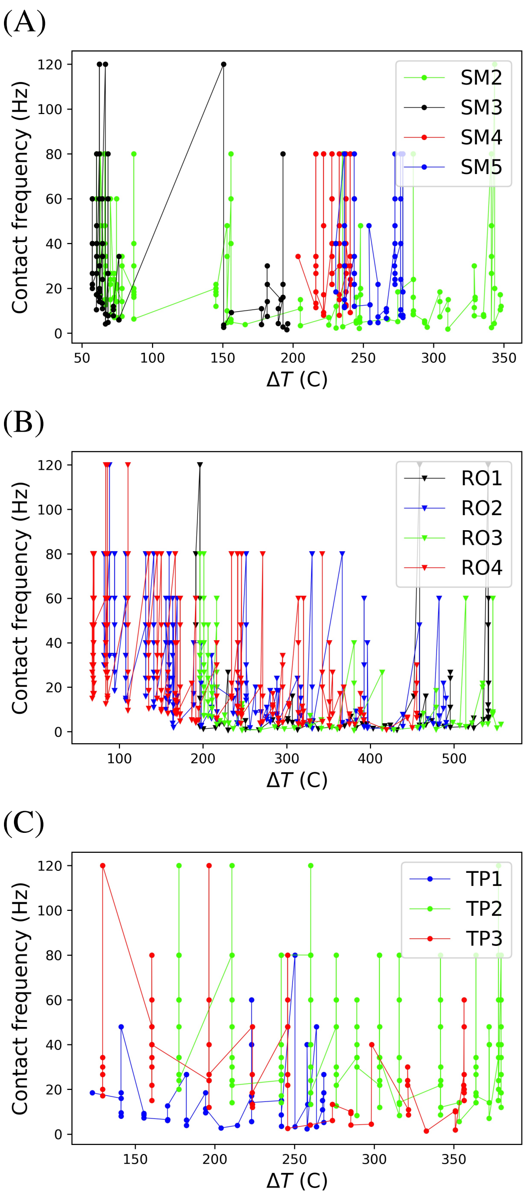

Figure S12. Under-sampled frequency of liquid-solid contact. Contact is defined as $\delta_{lv}^{min} \leq 8$ pixels (smooth sphere $\sim 14.8\ \mu m$, rough sphere $\sim 15.2\ \mu m$, thermoprobe $\sim 28.5\ \mu m$), which corresponds to half the thickness of the liquid-vapor interface. (A) Smooth sphere. (B) Rough sphere. (C) Thermoprobe.
